# The NephroCheck bedside system for detecting stage 3 acute kidney injury after open thoracoabdominal aortic repair

**DOI:** 10.1038/s41598-023-38242-2

**Published:** 2023-07-09

**Authors:** Panagiotis Doukas, Jan Paul Frese, Thorsten Eierhoff, Gabriel Hellfritsch, Ben Raude, Michael J. Jacobs, Andreas Greiner, Alexander Oberhuber, Alexander Gombert

**Affiliations:** 1 Department of Vascular and Endovascular Surgery, University Hospital Aachen, RWTH Aachen University, Pauwelsstrasse 30, 52074 Aachen, Germany; 2 Department of Vascular Surgery, Charité—Universitätsmedizin Berlin, Berlin, Germany; 3 Department of Vascular and Endovascular Surgery, University Hospital Muenster, Münster, Germany

**Keywords:** Biomarkers, Cardiology, Health care, Medical research, Molecular medicine, Nephrology, Risk factors

## Abstract

Acute kidney injury (AKI) is a common complication after complex aortic procedures and it is associated with relevant mortality and morbidity. Biomarkers for early and specific AKI detection are lacking. The aim of this work is to investigate the reliability of the NephroCheck bedside system for diagnosing stage 3 AKI following open aortic surgery. In this prospective, multicenter, observational study,—https://clinicaltrials.gov/ct2/show/NCT04087161—we included 45 patients undergoing open thoracoabdominal aortic repair. AKI risk (AKIRisk-Index) was calculated from urine samples at 5 timepoints: baseline, immediately postoperatively and at 12, 24, 48, and 72 h post-surgery. AKIs were classified according to the KDIGO criteria. Contributing factors were identified in univariable and multivariable logistic regression. Predictive ability was assessed with the area under the receiver operator curve (ROCAUC). Among 31 patients (68.8%) that developed AKIs, 21 (44.9%) developed stage-3 AKIs, which required dialysis. AKIs were correlated with increased in-hospital mortality (*p* = .006), respiratory complications (*p* < .001), sepsis (*p* < .001), and multi-organ dysfunction syndrome (*p* < .001). The AKIRisk-Index showed reliable diagnostic accuracy starting at 24 h post-surgery (ROCAUC: .8056, *p* = .001). In conclusion, starting at 24 h after open aortic repair, the NephroCheck system showed adequate diagnostic accuracy for detecting the patients at risk for stage 3 AKIs.

## Introduction

Acute kidney injury is an underestimated issue that occurs in a relevant proportion of patients that undergo open elective and emergency thoracoabdominal aortic repair^1^. In short- and mid-term follow ups, AKIs are directly related to a poor outcome and reduced survival^2,3^. In the context of major surgery, the timely detection of an adverse outcome, like an AKI, enables the immediate initiation of specific treatment bundles. In clinical practise, an AKI diagnosis is based on patient urine output and serum creatinine levels. This established biomarker is disputed, due to its delayed increase, its non-specificity, and its low sensitivity^4^. However, over the years, the combination of two markers, tissue inhibitor of metalloproteinases 2 (TIMP2) and insulin-like growth factor-binding protein 7 (IGFBP7), has gained clinical acceptance for the early detection of AKIs. Currently, these markers are available as a bedside-testing kit, called NephroCheck^5,6^. This study aimed to assess the NephroCheck system for its ability to detect AKIs in fields of open aortic surgery.

## Materials and methods

### Study design and ethics

This prospective, multicentric, observational trial was reviewed and authorized by the Ethics Committees of three participating centres: University Hospital Aachen (EK010/19), Charité—University Hospital Berlin, and University Hospital Münster (2019-559-b-S). The study was registered at clinicaltrials.gov with the number NCT04087161. Written informed consent was obtained from all included patients, and the study was conducted in accordance with the Declaration of Helsinki and the STROBE criteria.

The minimum sample size for a power of 80% was calculated to be n = 29, taking into account the values for sensitivity and specificity of the test reported in a cohort of patients undergoing cardiac surgery (Sensitivity 92%, Specificity 81%)^7^ and given a prevalence of 29% for postoperative AKI in TAAA patients^8^. The cohort of this study included 45 patients that were scheduled for aortic procedures that required suprarenal cross-clamping, from January 2019 to December 2021. Exclusion criteria were: age under 18 years, pregnancy, and terminal chronic kidney disease that required dialysis. Data were retrieved on demographic characteristics, comorbidities, and laboratory parameters from digitally maintained medical records.

### Surgery

The standardized, surgical protocol for open reconstruction of the thoracoabdominal aorta has been previously described in the literature^9^. The protective measures to reduce organ ischemia include distal aortic perfusion with extracorporeal circulation, selective perfusion of the visceral arteries and renal perfusion with maximum 1 L 4 °C Custodiol (Dr. Franz Köhler Chemie, Bensheim, Germany) for both kidneys along with mild systemic hypothermia (32–33 °C). The mean arterial pressure was kept at 90 mmHg during the procedure. The use of a cerebrospinal fluid drainage and the peri-operative monitoring of motor evoked potentials are implemented routinely to attenuate neurological complications and spinal cord ischemia. Postoperatively, all patients were administered unfractionated heparin for anticoagulation. Patients requiring therapeutic dosing maintained a partial thromboplastin time range of 50–60 s, while the remaining individuals in the cohort received a daily dosage of 15,000 IU of unfractionated heparin. Furthermore, volume management during the initial postoperative phase involved the use of invasive hemodynamic monitoring. The goal during this period was to maintain a positive fluid balance. However, in cases where fluid overload and/or oliguria occurred, deresuscitation was implemented. This involved administering a maximum daily dose of 80 mg of furosemide to address the excessive fluid burden.

### Measurements

Urine samples were aspirated from each patient’s urine catheter at 6 time points: preoperatively, immediately postoperatively, and at 12, 24, 48, and 72 h postoperatively. The samples were immediately centrifuged (4 °C, 1000 rpm for 10 min), and the supernatants were shock-frozen at − 80 °C. The NephroCheck system was applied, according to the manufacturer’s recommendations, to measure the concentrations of TIMP2 and IGFBP7. Then, the product of the concentrations of both proteins was calculated as AKIRisk Index, as follows: [TIMP2 × IGFBP7]/1000 (mg/L)^2^. The physicians involved in patients’ care were not aware of the results.

### Endpoints

The primary endpoint of this study was the association of AKIRisk-Index with the onset of AKIs requiring dialysis (AKI stage 3 of the KDIGO classification) after open aortic surgery. Indicators for continuous renal replacement therapy were: anuria, severe hyperkaliaemia (K^+^ > 6.5 mmol/L), severe metabolic acidosis (pH ≤ 7.2) and refractory volume overload^10^. The patients fulfilling these criteria received continuous veno-venous hemodialysis and are further referred to as the Stage 3 AKI group.

Secondary endpoints were the identification of risk factors that influenced the development of AKIs and postoperative complications other than AKIs. We tested the following risk factors: demographic characteristics, comorbidities, and procedural details. Postoperative complications were examined for correlations with postoperative dialysis treatment.

### Definitions

Bleeding was defined as a rapid decrease in hemoglobin levels by at least 3 g/dL within a 24-h period, substantiated by CT-angiographic verification of ongoing blood loss or the presence of a hematoma. Massive transfusion was defined as a transfusion of at least 10 blood bags within a 24-h period^11^. Patients were considered septic, when an infection was suspected, and their sequential organ failure assessment score increased by ≥ 2 points^12^. Acute liver failure was defined as the acute onset of jaundice and compromised synthetic function of the liver, based on a spontaneous international normalized ratio > 1.5, without a history of liver disease^13^. A multi-organ dysfunction syndrome (MODS) was diagnosed in patients with failure of two or more vital organ systems^14^. Acute respiratory distress syndrome (ARDS) was diagnosed according to the Berlin definition. It was classified in three categories, depending on the level of arterial hypoxemia: mild (ARDS 1), moderate (ARDS 2), and severe (ARDS 3)^15^. The severity of an AKI was classified according to the Kidney Disease Improving Global Outcomes criteria^16^: mild AKI was diagnosed in case of an increase of serum creatinine over 0.3 mg/dL within 48 h or 150% of baseline levels or if urine output was less than 0.5 mL/kg/h for 6 h. Further increase of serum creatinine up to 3 times the baseline level was considered moderate AKI and severe AKI was diagnosed in cases with a creatinine increase over 3 times the baseline level or urine output of less than 0.3 mL/kg/h for over 24 h or anuria for at least 12 h.

### Statistics

Continuous variables are reported as the mean (± standard deviation) or, in cases of heavily skewed distributions, as the median (25th–75th percentile limits). Categorical variables are presented as the frequency (n) and percentage. Significance was defined as a *p* value < 0.05 and *p* < 0.01; *p* values are expressed with the 95% confidence interval (CI). For categorical variables, the odds-ratio (OR) was calculated to evaluate risk factors, including patient demographics, comorbidities, and procedural details. For continuous variables, significant correlations were assessed with a univariable logistic regression analysis corrected with Firth’s bias correction; these models used the onset of AKI as the dependent variable. Univariable logistic regression analyses, corrected with the Firth’s bias correction, were also performed to examine associations between the development of AKI and the AKIRisk-index, daily urine output, serum creatinine level, and other postoperative complications.

The multivariable logistic regression model was adjusted with variables that were clinically known to correlate with AKI. These variables were related to patient’s comorbidities and the 24-h postoperative AKIRisk-Index. The dependent outcome was AKI development.

Receiver operating characteristic (ROC) curves were used to assess the ability of the AKIRisk-Index to predict postoperative dialysis treatment. The area under the ROC curve (ROC AUC) was calculated to evaluate the diagnostic value of the AKIRisk-Index measured at different time points. The cut-off value was selected for each time point with a prerequisite sensitivity (Se) of > 80%. The specificity (Sp) and likelihood ratio (LR) were adjusted accordingly. Statistical analyses were performed with SPSS software (SPSS Inc., Chicago IL). Graphs were designed with SPSS software and GraphPad Prism version 8.0.0 for Windows (GraphPad Software, San Diego, CA).

## Results

The presented cohort comprised 45 patients, including 17 women (37.7%), with a mean age of 58.4 ± 12.7 years. Details of patient comorbidities and procedural parameters are shown in Tables 1 and 2, respectively. 19 patients were previously diagnosed with chronic kidney disease with eGFR < 60 mL/min/1.73 m^2^ (according to the KDIGO classification: 9 patients were in stage G3a, 5 in stage G3b and 6 in G4). 2 patients in the cohort had preoperative creatinine levels over 2 mg/dL—2.5 and 2.3 mg/dL. The most common aortic procedure was an open reconstruction for a thoracoabdominal aortic aneurysm (TAAA), Crawford type III (n = 14; 31.1%). The next most common procedures were Crawford type II (n = 8; 17.8%) and type IV (n = 7; 15.6%) TAAA reconstructions (Supplementary Fig. 1). According to the KDIGO criteria for AKI, 13 patients (29%) developed an AKI based solely on urine output -oliguria or anuria- and 31 patients (69%) satisfied the diagnostic criteria based on the dynamic change of creatinine in serum. Mild AKIs were observed in 7 patients (15.6%). Three patients (6.7%) developed moderate AKIs, which were successfully treated with conservative management and fluid resuscitation. All patients with a stage 3 AKI (n = 21, 44.9%) required dialysis treatment (Table 3). Among these, the clinical criteria for the AKI diagnosis were met during the first postoperative day, for 9 patients (25.6%), and on the 11th postoperative day, for the last (21st) patient.

**Table 1:** Correlations between the onset of stage 3 AKI and the demographics and comorbidities of patients scheduled for aortic surgery.

Patients	Total, n = 45	Stage 3 AKI, n = 21	No stage 3 AKI, n = 24	Correlations	*p* value
Age (years)	58.4 ± 12.7	57 ± 15.3	59.8 ± 10	0.111 [− 0.39–0.19]†	0.469
Women	17 (37.7)	7 (33.3)	10 (41.7)	10.429 [0.42–4.83]	0.575
Obesity	6 (13.3)	3 (14.3)	3 (12.5)	1.167 [0.21–6.51]	0.864
Body mass index (kg/m^2^)	24.6 ± 4.7	23.7 ± 4.7	25.4 ± 4.6	0.184 [−0.45–0.12]†	0.227
Current smokers	18 (36)	7 (33.3)	11 (45.8)	0.500 [0.15–1.68]	0.269
Chronic obstructive pulmonary disease	10 (22.2)	4 (19)	6 (25)	0.706 [0.17–2.95]	0.641
Hypertension	33 (73.3)	18 (85.7)	15 (62.5)	3.600 [0.82–15.74]	0.082
Diabetes mellitus type II	3 (6.7)	2 (9.5)	1 (4.2)	2.421 [0.20–28.8]	0.484
Heart failure	9 (20)	4 (19)	5 (20.8)	0.894 [0.20–3.88]	0.885
Atrial fibrillation	4 (8.9)	0 (0)	4 (16.7)	0.488 [0.36–.067]	0.051
Myocardial infarction, history	5 (11.1)	2 (9.5)	3 (12.5)	0.737 [0.11–4.9]	0.758
Metallic aortic valve	4 (8.2)	2 (9.5)	2 (8.3)	1.158 [0.15–9.03]	0.892
Marfan syndrome	4 (8.2)	3 (14.3)	1 (4.2)	4.909 [0.45–53.27]	0.170
Stroke, history	5 (11.1)	3 (14.3)	2 (8.3)	1.833 [0.28–12.19]	0.537
Chronic kidney disease (eGFR < 60 ml/min/1.73 m^2^)	19 (42.2)	12 (57.1)	7 (29.2)	3.238 [0.94–11.12]	0.06
Aortobifemoral graft	12 (26.7)	5 (23.8)	7 (29.2)	0.759 [0.20–2.89]	0.693
Anticoagulation	13 (28.9)	4 (19)	9 (37.5)	0.392 [− 1–1.54]	0.18

Values are the number (%) or mean ± SD, as indicated. *Correlations are reported with the odds ratio and 95% confidence interval for categorical variables; however, those indicated with † are continuous variables, which are reported with Spearman’s (r) and the 95% confidence interval.

**Table 2:** Correlations between the onset of AKI and procedural parameters.

Patients	N = 45	Stage 3 AKI, n = 21	No stage 3 AKI, n = 24	Correlations*	*p* value
Occult rupture	5 (11.1)	3 (14.3)	2 (8.3)	0.583 [0.11–3.16]	0.537
Duration of procedure (min)	436 ± 146	474.9 ± 103	401.3 ± 169.4	0.255 [− 0.005–0.051]†	0.182
Aortic diameter (cm)	6.1 ± 1.8	6.6 ± 2.5	5.7 ± .9	0.225 [− 0.11–0.51]†	0.103
Crawford type I	4 (8.9)	1 (4.8)	1 (4.2)	3.833 [0.37–40.02]	0.244
Crawford type II	8 (17.8)	5 (23.8)	3 (12.5)	2.188 [0.45–10.54]	0.333
Crawford type III	14 (31.1)	9 (42.9)	5 (20.8)	2.850 [0.77–10.57]	0.116
Crawford type IV	7 (15.6)	2 (9.5)	5 (20.8)	0.400 [0.07–2.32]	0.307
Crawford type V	1 (2.2)	0 (0)	1 (4.2)	0.958 [0.88–1.04]	0.355
Juxtarenal AAA	6 (13.3)	0 (0)	6 (25)	0.750 [0.6–0.95]	0.013*
Aortic Stenosis	3 (6.7)	2 (9.5)	1 (4.2)	2.421 [0.2–28.8]	0.484
Aorto-oesophageal fistula	2 (4.4)	0 (0)	2 (8.3)	0.917 [0.81–1.03]	0.184

Values are the number (%) or mean ± SD, as indicated. *Correlations are reported with the odds ratio and 95% confidence interval for categorical variables; however, those indicated with † are continuous variables, which are reported with Spearman’s (r) and the 95% confidence interval.

*AAA* abdominal aortic aneurysm.

**Table 3:** AKIRisk-Index values [in (mg/dL)^2^] measured at different times before and after aortic surgery in patients that did or did not develop acute kidney injuries (AKIs).

Time points	No AKI (N = 14)	AKI stage 1(N = 7)	AKI stage 2 (N = 3)	AKI stage 3 (N = 21)
Preoperative	0.37 (0.19–0.83); *p* = .62	0.2 (0.1–0.34); *p* = 0.29	0.21 (0.16–0.21)^†^; *p* = 0.33	0.31 (0.16–0.76); *p* = 0.55
Directly postoperative	0.72 (0.17–2.7); *p* = .52	0.17 (0.07–0.78); *p* = 0.23	0.09 (0.08–0.09)^†^; *p* = 0.99	0.59 (0.3–1.45); *p* = 0.9
12 h	0.91 (0.35–2.17); *p* = 0.44	0.18 (0.15–0.87); *p* = 0.13	0.1 (0.1–0.1)^†^; *p* = 0.39	1.71 (0.38–3.15); *p* = 0.03*
24 h	0.78 (0.29–1.55); *p* = 0.15	0.31 (0.08–1.1); *p* = 1.3	2.74 (0.19–2.74)^†^; *p* = 0.82	3.71 (1.06–6.57); *p* = 0.004
48 h	0.62 (0.16–0.92); *p* = 0.04*	.2 (0.08–0.23); *p* = 0.13	1.57 (0.18–1.57)^†^; *p* = 0.39	4.79 (2.21–11.2); *p* < .001
72 h	0.81 (0.22–1.8); *p* = 0.03*	2.7 (0.13–2.7)^†^; *p* = 0.72	0.79 (0.13–0.79)^†^; *p* = 0.68	8.64 (7.19–23.86); *p* < .001

Values are the median (25th-Percentile–75th-Percentile), except those marked with (^†^), which are median (25th–50th-Percentile).

### Correlation between the AKIRisk-Index and AKI onset

**Figure 1:**
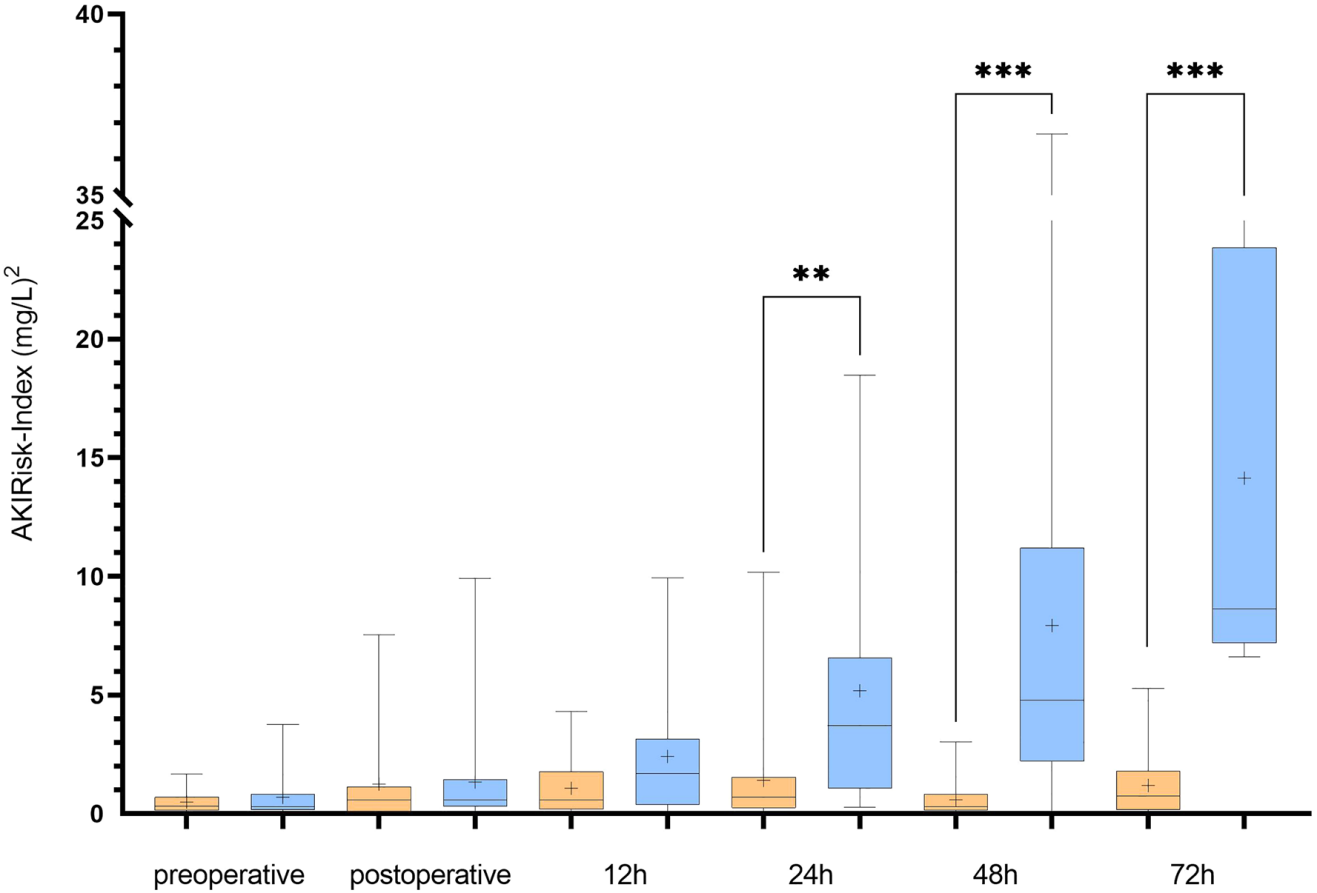
Box plot shows the AKIRisk-Index measured at different time points after aortic surgery. Orange: patients without AKIs, Blue: patients that developed stage 3 AKIs postoperatively. Boxes represent the interquartile range and median (horizontal line), whiskers represent minimum and maximum values. Sample means are marked with (+). Significant differences are marked with (**) for *p* < .01 and (***) for *p* < .001.

The AKIRisk-Index values were significantly correlated with the prevalence of postoperative stage 3 AKIs, starting from the 12-h time point after ICU admission (Table 3 and Fig. 1). We did not observe any significant association between AKIRisk-Index and AKI stage 1 or stage 2.

The 72-h course of AKIRisk-Index showed dynamics similar to those observed in the course of the clinical parameters currently used for assessing AKI development—creatinine serum levels and daily urine output (Supplementary Table 1). Serum creatine levels and the AKIRisk-Index were also similar in the significant correlation with the onset of AKI stage 3, as early as 24 h after surgery. The daily urine output was inversely correlated with the outcome, but the correlation was not as strong as those observed with serum creatinine and the AKIRisk-Index.

### Correlations between AKIs and postoperative complications

We constructed a univariable logistic regression model with the Firth’s bias correction to analyse correlations between postoperative dialysis treatment and other adverse outcomes (Table 4). We found that 42.9% of patients that developed stage 3 AKIs died before discharge. The intensive care unit (ICU) stay was significantly longer in the stage 3 AKI group than in the rest of the patients (34 days vs. 8 days, *p* = 0.009). We also found significant correlations between the onset of AKI requiring dialysis and respiratory complications, including pneumonia (n = 16 vs. 5, *p* < 0.001), duration of invasive ventilation (529 vs. 10.5 h; *p* < 0.001), and severe ARDS (n = 10 vs. 1, *p* < 0.001). In the stage 3 AKI group, 9 patients developed sepsis (42.9%), 8 were diagnosed with MODS (38.1%), and 7 were diagnosed with acute liver failure (33.3%). Patients that did not require dialysis did not develop any of these complications. The stage 3 AKI group experienced significantly higher frequencies of mass blood product transfusions (n = 18 vs. 8, *p* < 0.001), pulmonary bleeding (n = 7 vs. 0, *p* < 0.001), and revision thoracotomies (13 vs. 3, *p* < 0.001), compared to the rest of the patients.

**Table 4:** Correlations* between postoperative complications and stage 3 AKI onset after aortic surgery.

Patients	N = 45 (%)	Stage 3 AKI, n = 21	No stage 3 AKI, n = 24	*p* value [95% CI]
In-hospital mortality	11 (24.4)	9 (42.9)	2 (8.3)	0.006 [0.12–0.62]
Days to death	16 [3–58]	4.7 ± 6.4	5.7 ± 7	0.73 [− 0.47–0.35]
ICU stay	14 [6–34]	34 [10–51]	8 [6–16]	0.009 [0.1–0.61]
Hospital stay	32 [16–50]	37 [19–62]	21 [16–36]	0.12 [− 0.07–0.49]
Re-Thoracotomy	16(35)	13 (61,9)	3 (12,5)	< 0.001 [0.25–0.7]
Re-Laparotomy	4 (8.9)	2 (9.5)	2 (8.3)	0.89 [− 0.28–0.31]
Bleeding				
Abdominal	3 (6.7)	3 (14.3)	0 (0)	0.057 [− 0.12–0.53]
Intracranial	4 (8.9)	3 (14.3)	1 (4.2)	0.24 [− 0.13–0.45]
pulmonary	7 (15.6)	7 (33.3)	0 (0)	< 0.001 [0.26–0.7]
Mass transfusion	26 (59.1)	18 (85.7)	8 (33.3)	< 0.001 [0.26–0.7]
packed red blood cells (bags)	11 [5–26]	26 [16–30]	7 [2–10]	< 0.001 [0.52–0.83]
platelet concentrate (bags)	4 ± 4.7	6.7 ± 5.6	1.7 ± 1.9	< 0.001 [0.27–0.71]
FFP (bags)	15 [.5–26]	26 [21–41]	2,5 [0–14.25]	< 0.001 [0.4–0.77]
Fibrinogen (g)	3 ± 3.6	4.9 ± 4.2	1.5 ± 2	0.001 [0.2–0.67]
Sepsis	9 (20)	9 (42.9)	0 (0)	< 0.001 [0.28–0.71]
MODS	8 (17.8)	8 (38.1)	0 (0)	< 0.001 [0.23–0.69]
Acute liver failure	7 (15.6)	7 (33.3)	0 (0)	0.02 [0.19–0.66]
AKI 1	7 (15.6)			
AKI 2	3 (6.7)			
AKI 3 /Dialysis	21 (44.9)			
Time to AKI diagnosis (days)	1 [1, 2]			
Invasive ventilation (h)	16 [9–560]	529 [130–1158]	10.5 [5.2–17.5]	< 0.001 [0.38–0.77]
Re-Intubation	8 (17.8)	4 (19)	4 (16.7)	0.84 [− 0.27–0.32]
Pneumonia	21 (46.7)	16 (76.2)	5 (20.8)	< 0.001 [0.31–0.73]
ARDS 1	3 (6.7)	1 (4.8)	2 (8.3)	0.64 [− 0.36–0.23]
ARDS 2	4 (8.9)	4 (19)	0 (0)	0.025 [0.04–0.57]
ARDS 3	11 (24.4)	10 (47.6)	1 (4.2)	< 0.001 [0.24–0.7]
Apoplexy	5 (11.1)	4 (19.0)	1 (4.2)	0.12 [− 0.06–0.49]
Spinal cord ischaemia	5 (11.1)	3 (14.3)	2 (8.3)	0.54 [− 0.21–0.38]
Vocal cord paresis	3 (6.7)	2 (9.5)	1 (4.2)	0.48 [− 0.19–0.39]

*AKI* acute kidney injury, *ICU* intensive care unit, *FFP* fresh frozen plasma, *MODS* multi-organ dysfunction syndrome, *ARDS* acute respiratory distress syndrome.

*Correlations were calculated with a univariable, logistic regression model and Firth’s bias correction.

### Predictive value of the AKIRisk-Index

**Figure 2:**
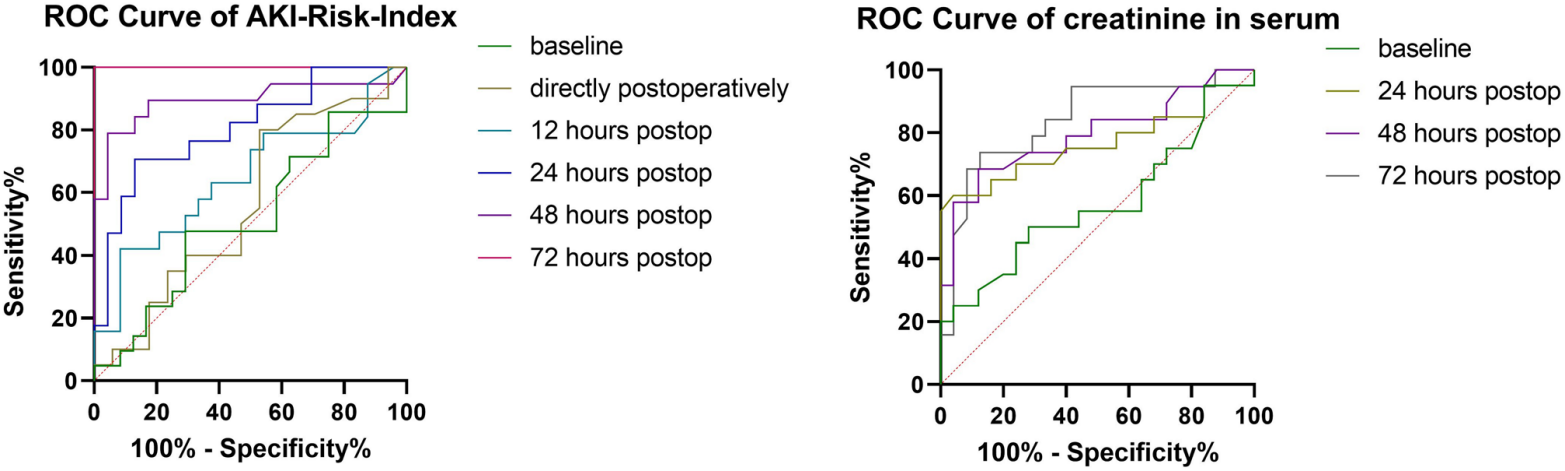
Receiver operating characteristic (ROC) curves for the AKIRisk-Index and serum creatinine levels show their abilities to predict stage 3 acute kidney injury (AKI) after aortic surgery. Postop: postoperative.

We investigated the diagnostic accuracy of the AKIRisk-Index with ROC-curve analyses (Table 5; Fig. 2). Preoperatively and immediately postoperatively, the test was not adequately informative (AUC: 0.51 and 0.56, respectively). Out of 25 patients with an AKIRisk-Index of 0.3 (mg/L)^2^ or greater preoperatively, 11 (44%) developed severe AKI after surgery. Starting at the 12-h time point, the test demonstrated some diagnostic ability (AUC 0.65, CI 0.48–0.82), but its value became excellent at the 24-h (AUC 0.80, CI 0.66–0.4), 48-h (AUC 0.89, CI 0.78–1), and 72-h (AUC 1, CI 1–1) time points. We calculated the optimal cut-off value for a sensitivity of at least 80%. Starting at the 12-h time point, a cut-off of > 0.14 (mg/L)^2^ was adequately sensitive, but at the cost of specificity (Se 84.21%, Sp 12.5%, LR 0.96). At the 24-h time point and later, the test’s accuracy and specificity improved, but the cut-off value had to be adjusted accordingly to 0.81 (mg/L)^2^. For the 12-h time point, when we set the cut-off value to > 0.3 (mg/L)^2^, we observed higher specificity and similar sensitivity (Se 78.95%, Sp 33.33%, LR 1.18; Supplementary table 2). Moreover, we found that increasing the cut-off value for the subsequent measurements provided adequate reliability without compromising accuracy.

**Table 5:** Receiver operating characteristic analysis results show the value of the AKIRisk Index and serum creatinine for predicting stage 3 acute kidney injuries (AKIs) after aortic surgery.

	Time point	AUC	95% CI	*p* value	Cut-off	Se (%)	Sp (%)	Likelihood ratio
AKIRisk Index	Preoperative	0.5149	0.34–0.69	0.87	> 0.15	80.95	25.00	1.079
	Postoperative	0.5647	0.37–0.76	0.50	> 0.26	80.00	47.06	1.511
	12 h	0.6480	0.48–.82	0.099	> 0.14	84.21	12.50	0.9624
	24 h	0.8056	0.67–0.95	0.001	> 0.81	82.35	56.52	1.894
	48 h	0.8947	0.78–1	< 0.001	> 1.23	84.21	86.96	6.456
	72 h	1.000	1–1	< 0.001	> 5.96	100	100	
Creatinine in serum	Preoperative	0.5740	0.4–0.75	0.40	> 0.77	85.00	16.00	1.012
	24 h	0.7640	0.61–0.92	0.003	> 1.36	80.00	44.00	1.429
	48 h	0.7958	0.65–0.94	< 0.001	> 1.32	84.21	52.00	1.754
	72 h	0.8410	0.72–0.97	< 0.001	> 1.31	84.21	66.67	2.526

*AKI* acute kidney injury, *AUC* area under the curve, *Se* sensitivity, *Sp* specificity.

As demonstrated above (Supplementary Table 1), the serum creatinine levels were significantly correlated with stage 3 AKI onset as soon as 24 h after surgery. However, the ROC curve analysis (Table 5, Fig. 2) revealed that the AKIRisk-Index showed better diagnostic accuracy than serum creatinine at all time points. The ROC-Curve analysis with the combination of creatinine in serum and urine output as predictors for stage 3 AKI, showed an AUC of 0.787 at 24 h, 0.775 at 48 h and 0.838 at 72 h, comparable to the AUCs of creatinine serum levels alone.

Next, we analysed the dataset of a subgroup that comprised the largest cohort (n = 23) recruited from one centre. We found that the AKIRisk-Index showed significant diagnostic efficiency as soon as 12 h after surgery (AUC 0.833, CI 0.65–1; Supplementary Table 3).

### Multivariable regression analysis

In a multivariable regression model, we assessed the predictive ability of AKIRisk-Index at 24 h after surgery—the earliest timepoint with significant diagnostic ability-, after adjusting for known clinical confounders for AKI (age, gender, chronic kidney disease and hypertension). The dependent variable for this model was AKI requiring RRT (Supplementary Table 4). The correlation of AKIRisk-Index with stage 3 AKI remained statistically significant at 24 h after adjusting for confounding.

## Discussion

This prospective, multicentre study confirmed that the NephroCheck bedside system was a feasible tool for detecting an AKI within the first hours after a major surgery, such as the open thoracoabdominal aortic repair. The current study was registered with an a priori protocol. Our findings were consistent with findings from previous studies that focused on AKIs after major surgery, including a systematic review^5,17^. In contrast, a recent trial on patients undergoing cardiac surgery could not confirm that the NephroCheck was beneficial for early postoperative AKI detection in the ICU^18^. However, those findings had limited transferability to our cohort, because the frequency of grade 3 AKI, which requires renal replacement therapy, was tenfold higher in our cohort than in that cohort (45.5% vs. 4%).

Mortality and morbidity are highly clinically relevant after open and endovascular thoracoabdominal aortic repair^9,19^. Consistent with previous trials that investigated larger cohorts^20^, we found a 24% mortality rate among patients undergoing open thoracoabdominal aortic repair. We also found that severe AKI was the most frequent complication after surgery, and it was significantly associated with other severe complications. For example, postoperatively, we observed clear correlations between AKIs and surgical revisions (mainly re-thoracotomy), bleeding complications, mass transfusions, sepsis, and multi-organ failure, again in accordance with the literature^1,21^. Furthermore, respiratory complications were the foremost complication: patients that required dialysis needed longer invasive ventilation times (560 h vs. 10.5 h), were more susceptible to pulmonary infections (76.2% vs. 20.8%), and were prone to hypoxaemia in the setting of severe ARDS (47.6% vs. 4.2%). Thus, timely identification of patients at-risk for AKI and the early initiation of preventive measures play an important role in patient’s recovery during the postoperative phase.

Serum creatinine and urine output are established markers of manifest kidney dysfunction, yet they may fail to accurately capture tubulointerstitial injury, before the impairment of the glomerular filtration rate becomes apparent^22^. Furthermore, confounding factors such as renal reserves, intravascular volume status and hemodynamic alterations may affect the changes in serum creatinine and urine output, limiting the accuracy of these parameters in diagnosing structural renal damage^23–25^. Specific renal biomarkers are able to detect tubular damage before changes in kidney function take place and thus enabling the diagnosis of AKI in its subclinical phase^26^. Their clinical applicability has been assessed in previous trials. For example, urinary neutrophil gelatinase associated lipocalin (NGAL) was correlated with postoperative AKIs that might require dialysis. However, the reliability of serum NGAL could not be confirmed^20^. A previous study validated the combination of TIMP2 and IGFBP7 for predicting AKIs in critically ill patients^7^. TIMP2 and IGFBP7 are biomarkers of cellular stress caused by inflammation and ischaemia in the early phase of tubular cell injuries. Indeed, these cytokines operate as paracrine signalling molecules when cellular damage occurs and promote the expression several tumor suppressor proteins like p53, p27 and p21, which in their turn inhibit cellular proliferation^27–29^. Keeping the cell cycle arrested at the G1 phase, is a temporary, energy-preserving, protective measure, which can end in fibrotic scaring of the organ tissue if prolonged^30^. In this notion, the NephroCheck bedside system calculates the AKIRisk-Index based on the concentrations of TIMP2 and IGFBP7 in patient urine and aims to detect this early warning signal of cellular stress, before manifest kidney dysfunction takes place.

Clinical evaluation of AKIRisk-Index has been investigated in several settings since the approval of NephroCheck by the Food and Drug Administration in September 2014. For patients undergoing cardiac surgery, the AKIRisk-Index reliably predicted the onset of AKI in the first postoperative day^31^ and was also found to be significantly, inversely correlated with kidney tissue recovery during follow-up^7^. Despite the growing body of evidence for the potential benefit of NephroCheck in perioperative setting, the inclusion of AKIRisk-Index measurements in diagnostic bundles has been met with scepticism by some physicians, who emphasize the need of further clinical validation^32^. For patients at risk for AKI after cardiac and abdominal surgery, early intervention and implementation of the KDIGO guidelines, as identified by biomarkers, has been shown to successfully attenuate the development of severe AKI and need for RRT^33,34^. Validated biomarkers, which accurately identify the patients, who may benefit from preventive interventions, are recommended in combination with clinical assessment in order to optimize the timing and type of interventions to improve patient outcomes^35^.

This was our motivation for assessing the NephroCheck in patients that underwent thoracoabdominal aortic repair. In this prospective, observational study, we found that the NephroCheck was a feasible tool for predicting postoperative AKI in 45 patients undergoing open aortic repair. It showed reliable predictive accuracy starting at 24 h after surgery. This finding was confirmed after adjusting for confounding factors. Indeed, the multivariable regression analysis confirmed that the postoperative NephroCheck measurement showed prognostic value in predicting AKIs (*p* = 0.01) after adjusting for age, gender, hypertension and chronic kidney disease.

None of the baseline characteristics of our cohort, correlated significantly with the onset of Stage 3 AKI. Surprisingly, pre-existing chronic kidney disease also did not significantly increase the postoperative AKI frequency in our cohort. Furthermore, none of the patients with juxtarenal AAA experienced stage 3 AKI postoperatively, although the renal perfusion protocol with 4 Custodiol solution remained the same. It should be noted, that a significant distinction between these patients and those with TAAAs was the shorter duration of cross-clamping time and the smaller extent of reconstruction in the juxtarenal AAA group. This findings emphasize the notion that the acute surgical trauma, with inflammation-reperfusion damage, was a relevant factor in the development of an AKI in this setting.

In our detailed analysis, both creatinine serum levels and a positive NephroCheck result were significantly correlated with the onset of AKI. However, a ROC-curve analysis revealed that the NephroCheck bedside system provided more reliable diagnostic accuracy than serum creatinine (AUC at 24 h: 0.806, *p* = 0.001 vs. 0.764, *p* = 0.003). Thus, our findings confirmed our working hypothesis, which was that the NephroCheck could be useful as an adjunct for diagnosing AKIs after open aortic surgery. When we focused on the large subgroup of patients from one centre, we observed that the NephroCheck had high diagnostic capacity, even at 12 h after surgery (AUC 0.833, Se 84.6%, Sp 77.8%). The increased accuracy in this subgroup, compared to the entire cohort, might be explained by differences in operative protocols and management strategies between the participating centres.

Recent studies established a cut-off value of > 0.3 (mg/L)^2^ as an AKIRisk-Index threshold for predicting AKIs; this value showed high sensitivity and a relatively low rate of false positives^36^. Preoperatively, we observed wide variability of the biomarkers’ concentrations, sometimes surpassing the 0.3 (mg/dL)^2^ threshold, which is in line with findings in current literature indicating susceptibility of the biomarkers to changes in urine concentration^37^. Our analysis confirmed the cut-off value of 0.3/mg/dL)^2^, but only for the 12-h measurement (Se 78.95%, Sp 33.33%, LR 1.18). At subsequent time points, we found that adjusting the cut-off value provided better specificity without impairing the test’s sensitivity.

This study had some relevant limitations. First, there was a small number of patients. This was partly due to the low numbers of thoracoabdominal aortic procedures performed world-wide annually. Moreover, this study was conducted between 2019 and 2021, during the COVID-19 pandemic; this situation caused serious inclusion issues for at least 2 years. Second, although we applied an a priori study protocol, several pre-, intra-, and postoperative parameters could not be standardized. For example, intraoperative decisions were based on the performing surgeon’s experience, and the postoperative course and ICU treatments were performed according to hospital standards. Indeed, despite the fact that we observed higher predictive accuracy when the NephroCheck was performed in a centre with a large variety of procedures, it is likely that a higher level of intra- and postoperative standardization would be favourable to its clinical reliability. Furthermore, given the susceptibility of AKIRisk-Index to changes in urine concentration, adjusting for urine osmolarity would support a more precise interpretation of the presented results^38^. Yet, the scope of this work was to investigate the practicability of the NephroCheck bedside system in clinical routine according to the manufacturer’s instructions, which do not recommend correction for dilution and, therefore, assessment of urine concentration was not included in our study protocol. Finally, although our study results were convincing, the methodology was strong, and the test quality was good, the analysis was conducted in a hypothesis-generating manner; thus, the findings require further clinical validation.

## Conclusion

In this prospective multicentre study, we confirmed the diagnostic accuracy of the NephroCheck bedside test for predicting postoperative AKIs that required dialysis in patients that underwent open thoracoabdominal aortic repair. We demonstrated that the test was accurate when performed within the first 24 h after surgery.

## Supplementary Information

Supplementary Information.

## Data Availability

The raw data supporting the conclusions of this article will be made available by the corresponding author (Panagiotis Doukas), without undue reservation.
