# Supplementary material for: The NephroCheck bedside system for detecting stage 3 acute kidney injury after open thoracoabdominal aortic repair
Source: Sci Rep. 2023 Jul 9;13:11096. doi: 10.1038/s41598-023-38242-2 (PMC10330159; doi:10.1038/s41598-023-38242-2)

The NephroCheck bedside system for detecting stage 3 acute kidney injury after open thoracoabdominal aortic repair

Panagiotis Doukas, MD[1*] –Jan Paul Frese, MD[2] – Thorsten Eierhoff, MD[3] – Gabriel Hellfritsch[1] – Ben Raude, MD[2] – Michael J. Jacobs, MD, PhD[1] – Andreas Greiner, MD, PhD[2] – Alexander Oberhuber, MD, PhD[3†] - Alexander Gombert, MD, PhD[1†]

[1]Department of Vascular and Endovascular Surgery, University Hospital Aachen, RWTH Aachen University, Germany

[2]Department of Vascular Surgery, Charité—Universitätsmedizin Berlin, Germany

[3]Department of Vascular and Endovascular Surgery, University Hospital Muenster, Germany

[†] These authors share last authorship

*** Correspondence:**
Panagiotis Doukas, MD; Pauwelsstrasse 30, 52074 Aachen, Tel: +49 (0) 241 80-80832, E-Mail: pdoukas@ukaachen.de

Supplementary Material:

Tables :5

Figures: 1

Supplementary Table 1: Correlations between the incidence of stage 3 acute kidney injury (AKI) and the daily urine output, serum creatinine, and AKI-Risk-Index.

Time point,	Parameter	Daily urine output	Serum creatinine	AKIRisk-Index
**Preoperative**	r		.249	.091
	p		.099	.55
	n		45	45
**Directly postoperative**	r			.022
	p			.9
	n			37
**12 h**	r			.324
	p			.034*
	n			43
**24 h**	r	-.255	.518	.445
	p	.094	.001**	.004**
	n	44	45	40
**48 h**	r	.02	.522	.524
	p	.9	p<.001***	p<.001***
	n	42	44	42
**72 h**	r	.132	.524	.779
	p	.41	p<.001***	p<.001***
	n	42	43	19

r: Spearman's correlation coefficient; p: p-value, calculated with a univariable logistic regression model and Firth's bias correction; AKI was the dependent variable; n= number of observations.

Supplementary Table 2: ROC analysis results for the predictive ability of the AKIRisk-Index in predicting stage 3 AKIs with a cut-off value of >.3 $(mg/L)^2$.

Time point	AUC	95% CI	P value	Cut-off	Se (%)	Sp (%)	Likelihood ratio
**Preoperative**	.5149	.34 - .69	.87	> .3050	52.38	41.67	.8980
**Postoperative**	.5647	.37 - .76	.50	> .3300	75.00	47.06	1.417
**12 h**	.6480	.48 - .82	.099	> .3	78.95	33.33	1.184
**24 h**	.8056	.67 - .95	.001	> .3550	88.24	34.78	1.353
**48 h**	.8947	.78 - 1	<.001	> .3050	89.47	52.17	1.871

ROC: receiver-operating characteristic; AKI: acute kidney injury; AUC: area under the curve; Se: sensitivity; Sp: specificity

Supplementary Table 3: ROC analysis results for the predictive ability of the AKIRisk-Index in predicting stage 3 AKIs in the subgroup that comprised the largest cohort (n=23) recruited from one centre.

Time point	AUC	95% CI	P value	Cut-off	Se (%)	Sp (%)	Likelihood ratio
**Preoperative**	.556	.31 - .81	.66	> .15	50.00	44.40	.89
**Postoperative**	.619	.32 - .87	.35	> .26	78.60	55.60	1.77
**12 h**	.833	.65 - 1	.009	> .14	84.60	77.80	3.81
**24 h**	.843	.66 - 1	.009	> .81	83.30	77.80	3.75
**48 h**	.910	.77 - 1	.001	> 1.23	84.60	77.80	3.81

ROC: receiver-operating characteristic; AUC: area under the curve; AKI: acute kidney injury; AUC: area under the curve; Se: sensitivity; Sp: specificity

Supplementary Table 4: Multivariable regression model results show the adjusted Odds-Ratios for clinical predictors of the risk for stage 3 AKI after aortic surgery.

	Adjusted Odds Ratio†	p-value
**AKIRisk-Index 24 h postoperatively, $(mg/dL)^2$**	1.05	.01*
**Hypertension, yes/no**	1.1	.62
**Chronic kidney disease, yes/no**	1.06	.7
**Age, years**	.99	.32
**Gender, male/female**	1.04	.79

*Significance for p<.05; †adjusted for age, gender, hypertension and chronic kidney disease

Supplementary Figure 1: Pie chart shows the frequencies of different aortic procedures among 45 patients that underwent aortic surgery. AAA: abdominal aortic aneurysm

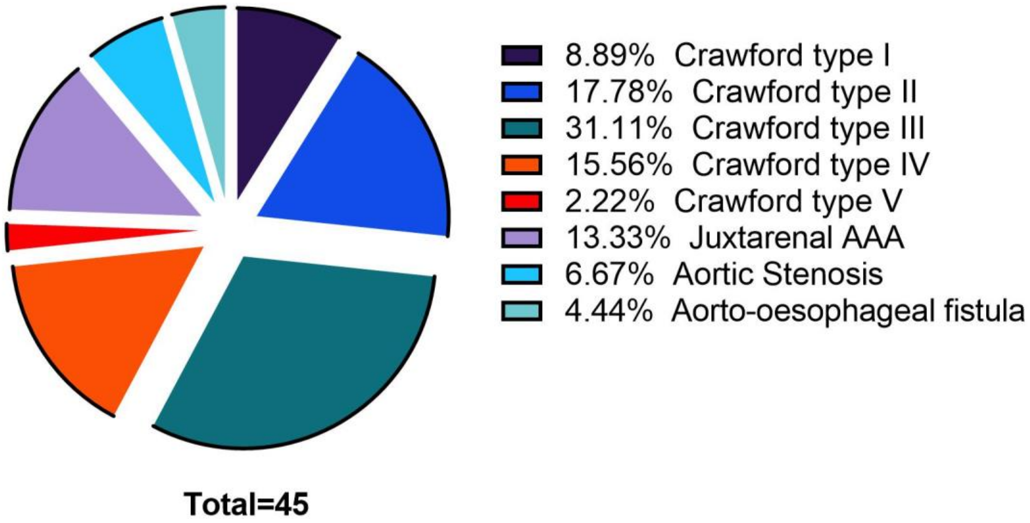

Supplement: Supplementary file 1 — Supplementary Information. [file 41598_2023_38242_MOESM1_ESM.pdf]
